# Differential practice and associated factors of COVID-19 personal preventive measures among the slum and estate communities of Uganda: A community-based cross-sectional survey

**DOI:** 10.7189/jogh.13.06039

**Published:** 2023-09-15

**Authors:** Joseph Kawuki, Joweria Nambooze, Paul Shing-fong Chan, Siyu Chen, Xue Liang, Phoenix K H Mo, Zixin Wang

**Affiliations:** 1 Centre for Health Behaviours Research, Jockey Club School of Public Health and Primary Care, The Chinese University of Hong Kong, Hong Kong, China; 2 Department of Nutritional Sciences and Dietetics, Kyambogo University, Kyambogo, Uganda

## Abstract

**Background:**

Compliance with personal preventive measures (PPMs) remains essential in the prevention and control of the coronavirus disease 2019 (COVID-19) pandemic and future infectious disease outbreaks. This study aimed at examining and comparing the practice of COVID-19 PPMs and associated factors in selected slum and estate communities of Uganda.

**Methods:**

This was a cross-sectional survey conducted among 1025 slum and estate residents in Uganda. The outcome variable was compliance with COVID-19 PPMs, including face mask use, hand washing/hygiene, and social distancing. Logistic regression models were fitted to assess the associated factors, using SPSS (version 26).

**Results:**

Of the 1025 participants, 511 and 514 were slum and estate residents, respectively. Compliance with PPMs was as follows; face mask use (slum 45.0% vs. estate 49.6%; *P* = 0.27), hand washing/hygiene (slum 38.4% vs. estate 44.9%; *P* = 0.04), and social distancing (slum 19.4% vs. estate 36.0%; *P* < 0.001). Compared to estate residents, slum residents had more knowledge related to COVID-19, perceived COVID-19 would have a longer timeline and larger impact on their life, had more depression and anxiety symptoms, and faced more difficulties to access information. Illness perceptions, infection risk, and severity perceptions were associated with higher odds of PPMs compliance in both groups, except for perceiving a high chance of contracting COVID-19, which was associated with lower odds of social distancing in the slum community. Depression and anxiety symptoms were associated with higher odds of PPMs compliance in both groups. Frequent exposure to COVID-19 information through health care workers and family members and friends was associated with higher odds of all the PPMs in both communities. Moreover, getting COVID-19 information from local channels was significantly associated with higher odds of mask use and hand hygiene, but only in the estate community.

**Conclusions:**

Our findings provided implications to improve PPMs compliance in future infectious disease outbreaks. To improve PPMs compliance rates, redesigning community education to focus on fostering positive perceptions and addressing the water and sanitation needs of slum communities are essential. Moreover, designing programs that provide free or subsidised face masks and soap to the most vulnerable and engaging religious leaders are also vital strategies.

Worldwide, the coronavirus disease 2019 (COVID-19) pandemic still poses significant challenges for health systems [1]. As of 27 April 2023, over 686 million cases and 6.8 million deaths due to COVID-19 have been reported globally, with over 12.8 million cases and 258 000 deaths reported in Africa [2]. In Uganda, over 170 000 cases and 3600 deaths have been reported since the start of the pandemic [2]. The COVID-19 pandemic has disproportionately affected slum communities across countries [3]. Moreover, current evidence shows that slum-dwellers have a higher vulnerability to COVID-19 infection and morbidity compared to other individuals with better living environments and socioeconomic conditions [4,5].

Although several vaccines against COVID-19 have been developed, existing vaccine hesitancy, as well as the emergence of new viral strains re-echo the importance of non-pharmaceutical interventions, particularly individuals' precautionary practices [6,7]. Such personal preventive measures (PPMs) that have gained public interest include wearing face masks, hand washing / hygiene, and social distancing, and these have been adopted by countries worldwide to limit disease transmission and infections, especially in the early stages of the pandemic [8-10]. Practice and adherence to these PPMs have been a primary way to prevent viral transmissions and slow disease spread, and are recommended by the World Health Organization [11].

However, given the influence of socioeconomic status, the urban poor, including slum-dwellers, have poorer compliance with COVID-19 PPMs. They live in deprived settings, which are overcrowded, making social distancing hardly possible [12-14]. Residents of informal settlements also have poor access to fresh, clean water, making hand washing / hygiene a challenge [15]. With the low income exacerbated by the adverse economic impact of the pandemic, buying face masks is also an extra economic burden [13,14,16]. Given this vulnerability, consideration of the urban poor and promotion of COVID-19 PPMs is crucial, and a clear understanding of factors that may hinder adherence in this group is vital for targeted interventions in the control of the pandemic.

Despite the evident vulnerability of the urban poor to the pandemic and the attested association of COVID-19 PPMs practice with socioeconomic status, few studies have explored the practice of PPMs among this special group. In Uganda, where about 50% of the urban residents live in informal settlements, i.e., slums [17], most of the COVID-19 PPMs studies have focused on the general community or other groups [18-21]. This poses uncertainty about whether the findings apply to the disadvantaged urban poor. Nonetheless, only one epidemiological study, to our knowledge, has examined the practice of COVID-19 PPMs among the slum communities of Uganda [22]. The study of Ugandan slum dwellers noted that the deprived living conditions in informal settlements hinder adherence to preventive measures such as mask use, social distancing, and hand hygiene [22]. Moreover, these slum dwellers had limited knowledge and access to COVID-19 testing services. Although the study highlighted several obstacles to compliance with COVID-19 PPMs, it did not include higher income or socioeconomic status comparison groups to examine inequities in PPMs practice, nor did it explore the possible determinants. To address these limitations, we conducted this comparative analysis of slum dwellers with more economically-advantaged communities.

This study used the socioecological model, in which individual and interpersonal level factors were considered [23]. Besides the common individual factors such as age, education level, and income, among others, this study also considered illness perceptions using the Brief Illness Perception Questionnaire (B-IPQ) and mental health. Current evidence shows that illness perceptions inform and influence peoples' subsequent coping behaviours [24], and in this context, it would influence compliance with PPMs practice [25]. In addition, previous studies have reported mental distress to be associated with compliance with PPMs practice among the general public [26,27]. Among interpersonal factors, COVID-19 information exposure via various sources was also considered in this study, since access and exposure to correct COVID-19 information could positively influence people’s knowledge, perceptions and attitudes toward PPMs practice [28]. Such hypotheses have not been tested in slum communities.

This study therefore aimed at examining and comparing the practice of COVID-19 personal preventive measures and associated factors in the slum and estate communities of Uganda. The estate communities represent a comparable better-off sample since they have more appropriate housing and better living conditions [29]. Understanding the factors that influence adherence to PPMs' practices in these two groups is essential for guiding policy and programming as well as designing targeted responses toward control of the current and future pandemics. We hypothesised that; i) slum residents would have lower adherence levels to COVID-19 PPMs practice compared to estate residents in formal settlements, and ii) there would be a difference in the factors associated with PPMs practice in the two groups.

## METHODS

### Study design

**Figure 1:**
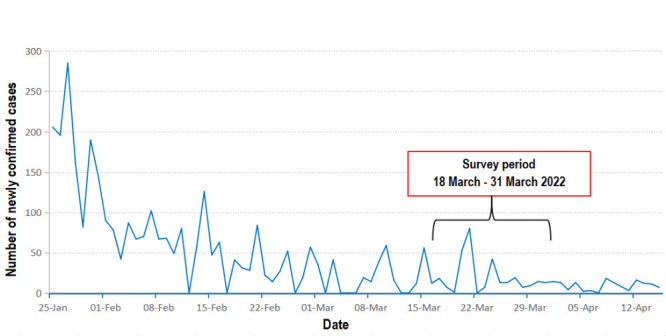
The COVID-19 situation in Uganda during the study period.

This was a cross-sectional survey among slum and estate communities conducted in Kampala and Wakiso districts in Uganda between March 18 and 31, 2022. Kampala is the capital city of Uganda, with 3.4 million residents, of whom about 50% live in informal settlements / slums [17]. Moreover, the city houses the largest urban slums in the country, has a population growth rate of 3.2% and accommodates 45% of all urban residents in Uganda [30]. Wakiso District is located in the Central Region of Uganda and partly encircles Kampala. Wakiso is the second wealthiest district in Uganda and houses most of the middle-class better-off urban residents of the country [31]. As shown in **Figure 1**, two peaks of daily COVID-19 cases were reported during the survey period; the first one on 21 March (80 cases) and the second recorded on 24 March (42 cases), after which daily cases remained stable and below 20 cases.

### Participants and sample size planning

The inclusion criteria included: 1) residents of slum communities or selected suburb estate communities, 2) aged 18 and above, and 3) could speak English or Luganda. Those who were not able to communicate effectively with the interviewers and those who lived in the study areas for less than six months were excluded.

Our target sample size was 1000 (500 from each group). We assumed the prevalence of compliance with PPMs to be 50.0% in the slum communities. Such a sample could detect the smallest difference of 8.8% in the PPMs compliance rate between the slum and estate communities, given a statistical power of 0.80 and an alpha value of 0.05 (two-sided) (PASS 11.0, NCSS LLC).

### Data collection

Details of the sampling and data collection methods were reported in a published study [32]. Ten out of all 57 slum communities in Kampala and Wakiso districts were randomly selected using the ballot method. Eligible households were identified using sampling frame mapping, after which systematic sampling was used to select the eligible households. Ten suburban estate communities in both Wakiso and Kampala districts were purposively selected for data collection. Approximately 50 households were randomly selected from each of the respective ten communities (20 in total). Trained interviewers approached each eligible household and invited one eligible household member to complete the interview. If there were more than one eligible household member available for the interview, the one whose last birthday was closest to the interview date was selected to respond to the survey. A similar approach was used in a previous population-based survey [33]. Written informed consent was sought.

**Figure 2:**
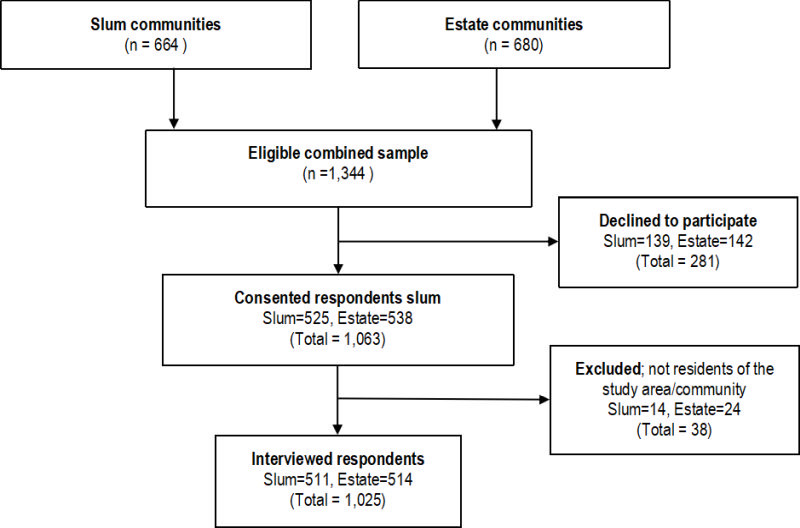
The flowchart diagram for participants' selection.

Face-to-face interviews in English and / or Luganda were conducted. The interview took about 30 minutes to complete. A total of 1344 households with eligible respondents were reached, of which 319 declined to participate or were excluded due to various reasons, resulting in a response rate of 76.3% (76.9% and 75.6% for slum and estate communities respectively), as shown in **Figure 2**. Ethics approval was obtained from the Clarke International University Institutional Review Board (CLARKE-2021-272) and the Survey and Behavioral Research Ethics Committee of the Chinese University of Hong Kong (SBRE-21-0148).

### Measures

#### Development of the questionnaire

A panel involving three public health researchers and a health psychologist was formed to develop the questionnaire. The English version of the questionnaire was translated into Luganda by bilingual researchers with master's degrees. The agreed-upon version was back-translated into English by independent bilingual researchers to ensure linguistic equivalence. The questionnaire was then pilot-tested among 27 slum and estate residents of Uganda to assess its clarity and appropriateness. From the pilot study, participants believed that the length was acceptable and the study contents were easy to understand. The 27 participants from the pilot test did not participate in the actual survey. The panel finalised the questionnaire.

#### Background characteristics

Background characteristics collected included sociodemographic characteristics (age, gender, education level, monthly household income, marital status, employment status, and religion), possession of electricity and piped water, whether the study participants shared toilets with other households, presence of chronic disease, and history of COVID-19 and COVID-19 vaccination.

#### Compliance with PPMs against COVID-19

We adapted validated measurements to assess compliance with three different PPMs [27,34]. They were: i) wearing a face mask when going to public places like workplaces, public transport, markets, shops, places of worship, etc; ii) washing hands with soap and clean water or sanitising hands using alcohol-based hand sanitisers; and iii) maintaining a reasonable social distance when in public places. The response categories to these PPMs were 1 = never, 2 = sometimes, 3 = often, and 4 = always. Similar to previous studies [27,34], compliance was considered if the respondent always practiced the PPMs.

#### Individual-level variables

Seven items were used to measure participants' knowledge about COVID-19. Six of these items were adapted from validated measurements in previous studies [34]. We added one item: “The most vulnerable group to severe COVID-19 are the elderly and those with chronic diseases.” The number of correct responses to these items was summed up, with a higher score indicating better knowledge of COVID-19.

Regarding perceptions about COVID-19, we assessed participants' representations of the disease using the Brief Illness Perception Questionnaire (B-IPQ) [35]. The scale was adapted by replacing “illness” in the original BIPQ with “COVID-19”. The original BIPQ has nine items, but in this study, we omitted the open-ended questions related to causality. The remaining eight BIPQ items measured: consequences, timeline, personal control, treatment control, identity, coherence, concern, and emotions related to COVID-19. The scores for each eight items ranged from 0 to 10, with higher scores indicating a more threatening perception of COVID-19. The B-IPQ has shown good test-retest reliability and validity in previous studies [21,35]. Additionally, participants' perceived susceptibility and severity of COVID-19 were also assessed with two validated items each.

Respondents’ mental health status was examined by considering the presence of depression symptoms and anxiety. We used the validated Patients' Health Questionnaire-9 (PHQ-9) scale and the Generalized Anxiety Disorder-7 (GAD-7) scale to measure depression and anxiety symptoms, respectively. The Cronbach's alpha of the PHQ-9 scale and the GAD-7 scale were 0.89 and 0.91, respectively.

#### Interpersonal-level variables

We measured participants' difficulty accessing COVID-19-related information using a 6-item scale constructed for this study (Cronbach's alpha: 0.89). The scale had questions on accessing COVID-19 information regarding signs and symptoms, statistics, treatment, personal preventive measures, vaccination, and government policies. We used validated items to explore the frequency of exposure to COVID-19-specific information through different channels, such as web-based media, local channels, health care workers, and family and friends [34].

#### Statistical analysis

The frequency distribution of all studied variables was presented. Differences in characteristics between the slum and estate groups were compared using either χ^2^ tests (for categorical variables) or independent-sample *t* tests (for continuous variables). Compliance with each of the three personal preventive measures (mask use, hand washing / hygiene, and social distancing) were the dependent variables. The differences in PPMs compliance and independent variables of interest were then compared using logistic or linear regression models, after controlling for background characteristics with significant between-group differences. Bivariate logistic regression models were then first fitted to assess the significance between background characteristics and the dependent variables in the two groups. A single multiple logistic regression model was then fitted, including all significant background characteristics and one independent variable of interest at a time. The crude odds ratios (OR), adjusted OR (AOR), and their 95% confidence intervals (CI) were obtained. Bivariate and multiple logistic regression models were also used to investigate the association between community memberships and dependent variables among all participants (combining slum and estate residents). We used SPSS 26.0 (IBM Corp., Armonk, NY, USA) for data analysis, with *P* < 0.05 as the level of statistical significance.

## RESULTS

### Characteristics of participants

The study consisted of 1025 participants, of which 511 were slum residents and 514 were estate residents. The majority of respondents were aged below 40 years (slum residents = 67.7%, estate residents = 62.4%), female (slum residents = 72.4%, estate residents = 69.5%), and married or cohabiting with a partner (slum residents = 61.4%, estate residents = 64.6%). In addition, the majority were employed full-time, part-time, or self-employed (slum residents = 67.5%, estate residents = 68.3%). Compared to estate residents, slum residents were younger (*P* = 0.001), more likely to have a monthly household income of Ugx 300 000 or below (68.9% vs. 28.8%; *P* < 0.001) and shared toilets with other households (91.8% vs. 77.2%; *P* < 0.001), but less likely to have secondary education or above (48% vs. 68.3%; *P* < 0.001), piped water (75.1% vs. 91.1%; *P* < 0.001) and electricity (90.0% vs. 95.5%; *P* < 0.001) in their households. Moreover, slum residents were, notably, more likely to have received at least one dose of COVID-19 vaccines (71.8% vs. 59.9%; *P* < 0.001), but less likely to have a confirmed COVID-19 infection history (8.0% vs. 13.0%; *P* = 0.01) compared to estate residents (**Table 1**).

**Table 1:** Background characteristics of the participants

	People from slum communities (n = 511)	People from estate communities (n = 514)	*P*-value
**Sociodemographic characteristics, n (%)**			
Age (years)			0.001
*18-29*	202 (39.5)	158 (30.7)	
*30-39*	144 (28.2)	163 (31.7)	
*40-49*	106 (20.7)	94 (18.3)	
*50 and above*	59 (11.5)	99 (19.3)	
Gender			0.16
*Male*	141 (27.6)	157 (30.5)	
*Female*	370 (72.4)	357 (69.5)	
Educational level			<0.001
*Primary or below*	266 (52.1)	163 (31.7)	
*Secondary*	193 (37.8)	207 (40.3)	
*Tertiary and above*	52 (10.2)	144 (28.0)	
Marital status			0.16
*Currently single*	197 (38.6)	182 (35.4)	
*Married or cohabiting with a partner*	314 (61.4)	332 (64.6)	
Current employment status			0.42
*Full-time/part-time/self-employed*	345 (67.5)	351 (68.3)	
*Unemployed/retired/student/housewife*	166 (32.5)	163 (31.7)	
Religion			0.73
*Catholic*	164 (32.1)	150 (29.2)	
*Protestant*	109 (21.3)	115 (22.4)	
*Moslem*	106 (20.7)	100 (19.5)	
*Pentecostal Christian*	118 (23.1)	132 (25.7)	
*Others*	14 (2.7)	17 (3.3)	
Monthly household income (Uganda Shillings*)			<0.001
*300 000 or below*	352 (68.9)	148 (28.8)	
*300 001-700 000*	133 (26.0)	216 (42.0)	
*700 001-3 000 000*	26 (5.1)	136 (26.5)	
*Above 3 000 000*	0 (0.0)	14 (2.7)	
Possess piped water in your household			<0.001
*No*	127 (24.9)	46 (8.9)	
*Yes*	384 (75.1)	468 (91.1)	
Possess electricity in your household			0.001
*No*	51 (10.0)	23 (4.5)	
*Yes*	460 (90.0)	491 (95.5)	
Sharing toilet with other households			<0.001
*No*	42 (8.2)	117 (22.8)	
*Yes*	469 (91.8)	397 (77.2)	
Household size			0.70
*5 and less members*	251 (49.1)	259 (50.4)	
*6 and above members*	260 (50.9)	255 (49.6)	
**Health conditions and COVID-19 vaccination status, n (%)**			
History of confirmed COVID-19 infection			0.01
*No*	470 (92.0)	447 (87.0)	
*Yes*	41 (8.0)	67 (13.0)	
Presence of chronic diseases			0.06
*No*	314 (61.4)	341 (66.3)	
*Yes*	197 (38.6)	173 (33.7)	
History of COVID-19 vaccination			<0.001
*Not vaccinated*	144 (28.2)	206 (40.1)	
*Got at least one dose*	367 (71.8)	308 (59.9)	

*1 US dollar = 3500 Uganda Shillings.

### Practice of personal preventive measures

Regarding compliance with personal preventive measures, slum residents were less likely to practice hand washing/hygiene (38.4% vs. 44.9%; *P* = 0.04), and maintain social distancing (19.4% vs. 36.0%; *P* < 0.001) compared to estate residents. In addition, they were less likely to wear face masks, although non-significant (45.0% vs. 49.6%; *P* = 0.27) (**Table 2**).

**Table 2:** Comparing compliance with personal preventive measures against COVID-19 and other independent variables of interest between people from slum communities and estate communities

	People from slum communities (n = 511)	People from estate communities (n = 514)	*P*-value	Adjusted *P*-value‡‡
**Compliance with personal preventive measures against COVID-19, n (%)**				
Wearing a face mask when going to public places like workplaces, public transport, market, shops, place of worship, etc.			0.08	0.22
*Never/sometimes/often*	281 (55.0)	259 (50.4)		
*Always*	230 (45.0)	255 (49.6)		
Washing hands with soap and clean water or sanitising hands using alcohol-based hand sanitisers			0.02	0.04
*Never/sometimes/often*	315 (61.6)	283 (55.1)		
*Always*	196 (38.4)	231 (44.9)		
Maintaining a reasonable social distance between you and others in public places			<0.001	<0.001
*Never/sometimes/often*	412 (80.6)	329 (64.0)		
*Always*	99 (19.4)	185 (36.0)		
Knowledge related to COVID-19, n (%)*				
COVID-19 can be transmitted through droplets of infected individuals	480 (93.9)	482 (93.8)	0.51	0.73
COVID-19 can be transmitted by touching contaminated objects/surfaces	473 (92.6)	478 (93.0)	0.44	0.52
COVID-19 can be transmitted through contact with asymptomatic patients	423 (82.8)	434 (84.4)	0.26	0.43
COVID-19 can be transmitted through contact with faeces	289 (56.6)	226 (44.0)	<0.001	<0.001
The most vulnerable group to severe COVID-19 are the elderly and those with chronic diseases	433 (84.7)	428 (83.3)	0.29	0.16
Some people can get infected with COVID-19 but have no signs and symptoms	387 (75.7)	383 (74.5)	0.35	0.80
Currently, there is no effective cure for COVID-19	207 (40.5)	197 (38.3)	0.26	0.02
Number of correct responses to knowledge related to COVID-19, mean (SD)	5.3 (1.3)	5.1 (1.3)	0.06	0.004
**Perceptions related to COVID-19**				
Brief illness representation (B-IPQ) related to COVID-19, mean (SD)				
*Consequences*	7.3 (3.1)	7.0 (3.0)	0.19	0.79
*Timeline*	5.5 (2.9)	5.1 (2.8)	0.02	0.04
*Personal control*	5.4 (3.3)	5.4 (3.1)	0.82	0.16
*Treatment control*	7.5 (2.6)	7.3 (2.4)	0.19	0.05
*Identity*	6.3 (2.6)	5.7 (2.7)	0.002	0.02
*Coherence*	7.2 (2.7)	7.3 (2.5)	0.39	0.75
*Concern*	8.9 (2.0)	8.8 (2.1)	0.49	0.12
*Emotions*	7.9 (2.7)	8.1(2.8)	0.36	0.08
Perceived susceptibility to COVID-19, n (%)†				
*In general, how high is your chance of contracting COVID-19 in the next 30 d?*	76 (14.9)	76 (14.8)	0.52	0.36
*How high is your chance of having close contact with people having COVID-19?*	121 (23.7)	109 (21.2)	0.19	0.23
Perceived severity of COVID-19, n (%)‡				
*COVID-19 would result in permanent bodily damage among infected people*	276 (54.0)	279 (54.3)	0.49	0.39
*People infected with COVID-19 have a high death rate*	346 (67.7)	351 (68.3)	0.45	0.06
**Mental health status**				
Depression symptoms (score of the PHQ-9 scale§), mean (SD)	5.8 (6.1)	5.0 (5.5)	0.02	0.001
Generalised anxiety symptoms (score of the GAD-7 scale‖), mean (SD)	5.1 (5.1)	4.5 (4.8)	0.07	0.03
**Difficult to access COVID-19-related information, n (%)**¶				
Signs and symptoms of COVID-19	103 (20.2)	84 (16.3)	0.07	0.48
COVID-19 statistics in Uganda	140 (27.4)	100 (19.5)	0.002	0.02
Treatment of COVID-19 in Uganda	127 (24.9)	105 (20.4)	0.05	0.84
COVID-19 personal preventive measures	59 (11.5)	64 (12.5)	0.36	0.01
COVID-19 vaccination programme in Uganda	73 (14.3)	65 (12.6)	0.25	0.39
Government policies concerning COVID-19	34 (6.7)	40 (7.8)	0.28	0.23
Difficult to access COVID-19 Information Scale**, mean (SD)	1.1 (1.6)	0.9 (1.6)	0.11	0.47
**Exposure to COVID-19-specific information through different channels, n (%)**††				
Web-based media	113 (22.1)	178 (34.6)	<0.001	<0.001
Local channels	340 (66.5)	316 (61.5)	0.05	0.01
Healthcare workers	227 (44.4)	230 (44.7)	0.48	0.41
Family members and friends	251 (49.1)	257 (50.0)	0.41	0.69

B-IPQ – brief illness perception questionnaire, SD – standard deviation, PHQ-9 – patient’s health questionnaire-9, GAD-7 – generalised anxiety disorder-7

*Correct response.

†High/very high.

‡Agree/strongly agree.

§PHQ-9 scale, 9 items; Cronbach's alpha: 0.89, one factor was identified by exploratory factor analysis, explaining 54.8% of the total variance.

‖GAD-7 scale, 7 items; Cronbach's alpha: 0.91, one factor was identified by exploratory factor analysis, explaining 64.6% of the total variance.

¶Difficult/very difficult.

**Difficult to access COVID-19 Information Scale, 6 items; Cronbach's alpha: 0.89, one factor was identified by exploratory factor analysis, explaining 64.2% of the total variance.

††Sometimes/always.

‡‡Adjusted for background variables with significant between-group differences.

### Descriptive statistics of independent variables of interest

The item responses and scale scores for knowledge, perceptions, mental health, difficulty in accessing COVID-19 information, and exposure to various information channels are detailed in **Table 2**. Compared to estate residents, more slum residents knew that COVID-19 can be transmitted through faeces (56.6% vs. 44.0%; *P* < 0.001), and currently, there is no effective cure for COVID-19 (40.5% vs. 38.3%; *P* = 0.01). Slum residents believed that COVID-19 would have a longer timeline (*P* = 0.04) and had a larger impact on their life (identity) (*P* = 0.02) compared to estate residents. In addition, slum residents had more depression (*P* = 0.001) and anxiety (*P* = 0.03) symptoms compared to the estate residents. They were also more likely to face difficulties in accessing information about COVID-19 statistics (27.4% vs. 19.5%; *P* = 0.02), and had less exposure to COVID-19-specific information through web-based media (22.1% vs. 34.6%; *P* < 0.001), (**Table 2**).

### Association of background characteristics with compliance with personal preventive measures against COVID-19 among slum and estate residents

Among the slum residents, higher odds of compliance with face mask use were among those with older age, higher household monthly income, and COVID-19 vaccination, but with lower odds among those who shared toilets with other households. Higher odds of compliance with hand hygiene were associated with older age and having piped water, while higher odds of social distancing were associated with older age, having piped water, and large household size (**Table 3**).

**Table 3:** Association of background characteristics with compliance with personal preventive measures against COVID-19 among people from slum and estate communities

Sociodemographic characteristics	Compliance with mask use				Compliance with hand washing/hygiene				Compliance with social distance			
	**People from slum communities (n = 511)**		**People from estate communities (n = 514)**		**People from slum communities (n = 511)**		**People from estate communities (n = 514)**		**People from slum communities (n = 511)**		**People from estate communities (n = 514)**	
	**UOR (95% CI)**	***P*-value**	**UOR (95% CI)**	***P*-value**	**UOR (95% CI)**	***P*-value**	**UOR (95% CI)**	***P*-value**	**UOR (95% CI)**	***P*-value**	**UOR (95% CI)**	***P*-value**
**Age (years)**												
18-29	Referent category		Referent category		Referent category		Referent category		Referent category		Referent category	
30-39	1.25 (0.81-1.93)	0.30	1.22 (0.78-1.90)	0.38	1.81 (1.16-2.82)	0.01	1.21 (0.77-1.90)	0.41	1.64 (0.93-2.88)	0.09	1.01 (0.63-1.61)	0.96
40-49	1.77 (1.10-2.85)	0.02	1.77 (1.06-2.97)	0.03	1.67 (1.02-2.71)	0.04	1.90 (1.13-3.20)	0.02	2.34 (1.30-4.20)	<0.001	1.08 (0.63-1.86)	0.77
50 and above	1.38 (0.77-2.47)	0.28	2.50 (1.49-4.20)	<0.001	1.66 (0.91-3.02)	0.10	3.05 (1.81-5.14)	<0.001	1.59 (0.75-3.36)	0.23	2.06 (1.23-3.45)	0.01
**Gender**												
Male	Referent category		Referent category		Referent category		Referent category	0.78	Referent category		Referent category	
Female	0.94 (0.64-1.39)	0.76	1.07 (0.74-1.56)	0.72	1.19 (0.79-1.78)	0.41	0.95 (0.65-1.38)		1.42 (0.85-2.39)	0.18	0.94 (0.64-1.39)	0.77
**Educational level**												
Primary or below	Referent category		Referent category		Referent category		Referent category		Referent category		Referent category	
Secondary	1.01 (0.70-1.47)	0.94	1.38 (0.91-2.09)	0.13	0.98 (0.67-1.44)	0.93	1.29 (0.84-1.96)	0.24	0.71 (0.44-1.15)	0.17	0.83 (0.54-1.29)	0.41
Tertiary and above	1.06 (0.58-1.92)	0.85	2.59 (1.63-4.11)	<0.001	0.91 (0.49-1.69)	0.77	2.76 (1.74-4.39)	<0.001	0.75 (0.35-1.63)	0.47	1.49 (0.94-2.36)	0.09
Monthly household income (Uganda Shillings*)												
300 000 or below	Referent category		Referent category		Referent category		Referent category		Referent category		Referent category	
300 001-700 000	1.52 (1.02-2.27)	0.04	0.99 (0.65-1.51)	0.98	1.26 (0.84-1.89)	0.27	1.28 (0.83-1.96)	0.26	1.25 (0.77-2.05)	0.37	0.73 (0.47-1.14)	0.17
700 001-3 000 000	1.92 (0.86-4.31)	0.11	2.14 (1.33-3.44)	<0.001	1.27 (0.57-2.84)	0.57	1.96 (1.22-3.15)	0.01	1.35 (0.52-3.50)	0.54	1.05 (0.65-1.69)	0.84
Above 3 000 000	N/A		1.24 (0.41-3.72)	0.70	N/A		4.23 (1.26-14.13)	0.02	N/A		1.20 (0.39-3.63)	0.75
**Marital status**												
Currently single	Referent category		Referent category		Referent category		Referent category		Referent category		Referent category	
Married or cohabiting with a partner	1.13 (0.79-1.62)	0.50	0.85 (0.59-1.22)	0.39	0.86 (0.59-1.23)	0.41	0.81 (0.56-1.16)	0.25	1.06 (0.68-1.67)	0.79	0.63 (0.44-0.92)	0.02
**Current employment status**												
Full-time/part-time/self-employed	Referent category		Referent category		Referent category		Referent category		Referent category		Referent category	
Unemployed/retired/student/housewife	0.87 (0.60-1.27)	0.48	1.00 (0.69-1.46)	0.98	0.75 (0.51-1.10)	0.14	0.86 (0.59-1.25)	0.42	1.17 (0.74-1.86)	0.50	1.01 (0.69-1.49)	0.95
**Religion**												
Catholic	Referent category		Referent category		Referent category		Referent category		Referent category		Referent category	
Protestant	1.15 (0.71-1.87)	0.57	0.65 (0.40-1.07)	0.09	0.94 (0.58-1.55)	0.82	1.03 (0.63-1.69)	0.89	0.76 (0.42-1.39)	0.38	0.72 (0.43-1.21)	0.22
Moslem	0.97 (0.59-1.58)	0.90	1.80 (1.07-3.01)	0.03	0.74 (0.45-1.24)	0.26	1.64 (0.99-2.73)	0.06	0.79 (0.44-1.44)	0.44	1.25 (0.75-2.08)	0.40
Pentecostal Christian	0.86 (0.54-1.39)	0.55	1.05 (0.66-1.68)	0.82	0.86 (0.53-1.40)	0.55	0.99 (0.62-1.59)	0.97	0.54 (0.29-1.01)	0.05	0.77 (0.47-1.25)	0.29
Others	1.22 (0.41-3.62)	0.73	1.19 (0.43-3.24)	0.74	1.09 (0.36-3.27)	0.88	0.73 (0.26-2.09)	0.56	0.25 (0.03-1.95)	0.18	0.49 (0.15-1.57)	0.23
**Possess piped water in your household**												
No	Referent category		Referent category		Referent category		Referent category		Referent category		Referent category	
Yes	1.19 (0.80-1.79)	0.39	1.08 (0.59-1.98)	0.80	1.64(1.06-2.52)	0.02	2.21 (1.14-4.31)	0.02	2.53 (1.36-4.71)	<0.001	1.48 (0.76-2.88)	0.25
**Possess electricity in your household**												
No	Referent category		Referent category		Referent category		Referent category		Referent category		Referent category	
Yes	1.30 (0.72-2.35)	0.38	1.56 (0.66-3.68)	0.31	1.16 (0.63-2.12)	0.64	1.28 (0.55-3.02)	0.57	1.14 (0.53-2.42)	0.74	0.60 (0.26-1.39)	0.23
**Sharing toilet with other households**												
No	Referent category		Referent category		Referent category		Referent category		Referent category		Referent category	
Yes	0.30 (0.15-0.60)	0.001	0.27 (0.17-0.42)	<0.001	1.01 (0.53-1.94)	0.97	0.35 (0.22-0.53)	<0.001	0.75 (0.36-1.58)	0.45	0.91 (0.60-1.40)	0.68
**Household size**												
5 and less members	Referent category		Referent category		Referent category		Referent category		Referent category		Referent category	
6 and above members	1.33 (0.94-1.89)	0.11	1.62 (1.15-2.30)	0.01	1.15 (0.81-1.65)	0.44	1.34 (0.95-1.91)	0.10	1.91 (1.21-3.00)	0.01	1.19 (0.83-1.71)	0.34
**Health conditions**												
History of confirmed COVID-19 infection												
*No*	Referent category		Referent category		Referent category		Referent category		Referent category		Referent category	
*Yes*	0.85 (0.45-1.63)	0.63	1.72 (1.02-2.90)	0.04	1.03 (0.54-1.98)	0.93	1.99 (1.18-3.35)	0.01	0.85 (0.36-1.97)	0.70	1.33 (0.79-2.24)	0.29
Presence of chronic diseases												
*No*	Referent category		Referent category		Referent category		Referent category		Referent category		Referent category	
*Yes*	1.23 (0.86-1.77)	0.25	1.38 (0.95-1.99)	0.09	0.82 (0.57-1.19)	0.30	1.34 (0.93-1.93)	0.12	1.50 (0.96-2.34)	0.07	1.44 (0.99-2.10)	0.06
**Vaccination status**												
Not vaccinated	Referent category		Referent category		Referent category		Referent category		Referent category		Referent category	
Got at least one dose	1.54 (1.03-2.28)	0.03	1.30 (0.92-1.86)	0.14	1.05 (0.71-1.57)	0.80	1.47 (1.02-2.10)	0.04	0.93 (0.58-1.52)	0.78	0.90 (0.63-1.31)	0.59

UOR – unadjusted odds ratios, CI – confidence interval, N/A – not available

*1 US dollar = 3500 Uganda Shillings.

Among estate residents, higher odds of face mask use compliance were associated with older age, tertiary education and above, higher household monthly income, Moslem religion, large household size, and confirmed COVID-19 infection history, but with less odds associated with toilet sharing. Higher odds of compliance with hand washing / hygiene were associated with older age, tertiary education and above, higher household monthly income, having piped water, a confirmed COVID-19 infection history, and COVID-19 vaccination, but with less odds associated with toilet sharing. Higher odds of compliance with social distancing among estate residents were associated with older age but with less odds associated with being married or cohabiting with a partner (**Table 3**).

### Association of variables of interest with compliance with personal preventive measures against COVID-19 among slum and estate residents

The unadjusted associations for the key variables of interest with compliance with personal preventive measures are detailed in Table S1 in the **Online Supplementary Document**. After adjusting for significant background characteristics, **Table 4** indicates that perceiving COVID-19 to have more consequences (AOR = 1.06; 95% CI = 1.00-1.13 and AOR = 1.10; 95% CI = 1.03-1.17), timeline (AOR = 1.13; 95% CI = 1.06-1.21 and AOR = 1.14; 95% CI = 1.06-1.23) emotional effect (AOR = 1.14; 95% CI = 1.06-1.22 and AOR = 1.10; 95% CI = 1.03-1.18), and having a more identity of the disease (both AOR = 1.12; 95% CI = 1.04-1.21) were associated with higher odds of compliance with face mask use in both slum and estate communities. Having more coherence (AOR = 1.09; 95% CI = 1.01-1.19) and concerns (AOR = 1.33; 95% CI = 1.17-1.50) about the disease were associated with higher odds of mask use only in the estate community. Perceiving having high chances of close contact with people having COVID-19 (AOR = 1.72; 95% CI = 1.07-2.79) was also associated with higher odds of mask use, but only in the estate community. Having depression symptoms (AOR = 1.04; 95% CI = 1.01-1.07 and AOR = 1.09; 95% CI = 1.05-1.13) was associated with higher odds of mask use in both communities while having anxiety symptoms (AOR = 1.08; 95% CI = 1.03-1.12) was associated with higher odds only in the estate community. Moreover, frequent exposure to COVID-19 information through health care workers (AOR = 1.47; 95% CI = 1.01-2.15 and AOR = 1.83; 95% CI = 1.22-2.74), and family members and friends (AOR = 1.73; 95% CI = 1.19-2.53 and AOR = 2.92; 95% CI = 1.93-4.43) were also associated with higher odds of mask use in both slum and estate communities. Getting COVID-19 information from local channels (AOR = 2.73; 95% CI = 1.78-4.20) was associated with higher odds of mask use but only in the estate community (**Table 4**).

**Table 4:** Adjusted association of variables of interest with compliance with personal preventive measures against COVID-19 among people from slum and estate communities

Variable	Compliance with mask use*				Compliance with hand washing/hygiene*				Compliance with social distance*			
	**People from slum communities (n = 511)**		**People from estate communities (n = 514)**		**People from slum communities (n = 511)**		**People from estate communities (n = 514)**		**People from slum communities (n = 511)**		**People from estate communities (n = 514)**	
	**AOR (95% CI)†**	***P*-value**	**AOR (95% CI)†**	***P*-value**	**AOR (95% CI)‡**	***P*-value**	**AOR (95% CI)‡**	***P*-value**	**AOR (95% CI)**§	***P*-value**	**AOR (95% CI)**§	***P*-value**
**Perceptions related to COVID-19**												
Brief illness representation (B-IPQ) related to COVID-19												
*Consequences*	1.06 (1.00-1.13)	0.04	1.10 (1.03-1.17)	0.01	1.04 (0.98-1.11)	0.21	1.04 (0.98-1.11)	0.19	1.06 (0.98-1.14)	0.14	1.03 (0.97-1.10)	0.32
*Timeline*	1.13 (1.06-1.21)	<0.001	1.14 (1.06-1.23)	<0.001	1.10 (1.03-1.17)	0.004	1.10 (1.02-1.17)	0.01	1.06 (0.98-1.14)	0.16	1.06 (0.99-1.13)	0.08
*Personal control*	1.01 (0.95-1.07)	0.80	1.00 (0.94-1.07)	0.98	1.01 (0.95-1.07)	0.79	0.95 (0.89-1.01)	0.12	0.95 (0.89-1.02)	0.18	0.94 (0.89-1.00)	0.06
*Treatment control*	1.03 (0.96-1.10)	0.48	1.04 (0.96-1.13)	0.35	1.02 (0.95-1.09)	0.56	1.01 (0.94-1.10)	0.72	0.97 (0.90-1.06)	0.55	1.04 (0.96-1.12)	0.34
*Identity*	1.12 (1.04-1.21)	0.002	1.12 (1.04-1.21)	0.002	1.10(1.03-1.19)	0.01	1.01 (0.94-1.09)	0.69	1.05(0.96-1.14)	0.29	1.06 (0.99-1.13)	0.11
*Coherence*	1.04 (0.97-1.11)	0.33	1.09 (1.01-1.19)	0.03	0.96(0.90-1.03)	0.28	1.01 (0.94-1.10)	0.75	0.99(0.91-1.08)	0.88	1.02 (0.95-1.10)	0.59
*Concern*	0.99 (0.90-1.09)	0.90	1.33 (1.17-1.50)	<0.001	1.04 (0.94-1.14)	0.47	1.22 (1.09-1.36)	<0.001	0.98 (0.88-1.10)	0.74	1.20 (1.07-1.33)	0.001
*Emotions*	1.14 (1.06-1.22)	0.001	1.10 (1.03-1.18)	0.01	1.10 (1.03-1.19)	0.01	1.05 (0.98-1.13)	0.13	1.13 (1.03-1.24)	0.01	1.07 (1.00-1.15)	0.04
Perceived susceptibility to COVID-19‖												
*In general, how high is your chance of contracting COVID-19 in the next 30 d?*	0.76 (0.45-1.30)	0.32	1.42 (0.82-2.45)	0.21	1.11 (0.66-1.86)	0.70	2.03 (1.20-3.43)	0.01	0.27 (0.10-0.69)	0.01	1.34 (0.81-2.22)	0.25
*How high is your chance of having close contact with people having COVID-19*	1.33 (0.86-2.06)	0.20	1.72 (1.07-2.79)	0.03	1.62 (1.06-2.48)	0.03	2.28 (1.43-3.62)	0.001	0.92 (0.53-1.57)	0.75	1.39 (0.89-2.16)	0.15
Perceived severity of COVID-19¶												
*COVID-19 would result in permanent bodily damage among infected people*	0.78 (0.54-1.14)	0.21	1.47 (0.99-2.18)	0.06	0.96 (0.66-1.38)	0.82	1.06 (0.73-1.56)	0.75	1.55 (0.97-2.46)	0.07	1.48 (1.02-2.15)	0.04
*People infected with COVID-19 have a high death rate*	1.05 (0.70-1.57)	0.81	1.10 (0.71-1.70)	0.68	1.17 (0.79-1.74)	0.43	1.10 (0.73-1.68)	0.64	2.03 (1.18-3.49)	0.01	1.35 (0.90-2.01)	0.15
**Knowledge related to COVID-19**												
Number of correct responses to knowledge related to COVID-19	1.01 (0.88-1.17)	0.87	1.11 (0.96-1.29)	0.17	1.00 (0.87-1.15)	0.97	1.02 (0.89-1.18)	0.76	0.93 (0.79-1.10)	0.41	1.12 (0.98-1.29)	0.10
**Mental health status**												
Depression symptoms (score of the PHQ-9 scale)	1.04 (1.01-1.07)	0.01	1.09 (1.05-1.13)	<0.001	1.03 (1.00-1.06)	0.04	1.07 (1.03-1.11)	<0.001	1.05 (1.02-1.09)	0.004	1.08 (1.05-1.12)	<0.001
Generalised anxiety symptoms (score of the GAD-7 scale)	1.03 (0.99-1.07)	0.17	1.08 (1.03-1.12)	0.001	1.02 (0.98-1.05)	0.41	1.04 (1.00-1.08)	0.03	1.05 (1.00-1.09)	0.04	1.08 (1.04-1.12)	<0.001
**Difficult to access COVID-19-related information**												
Difficult to access COVID-19 Information Scale	0.89 (0.79-1.01)	0.07	1.06 (0.94-1.21)	0.33	0.90 (0.79-1.01)	0.08	0.94 (0.83-1.06)	0.30	0.96 (0.83-1.11)	0.59	0.96 (0.86-1.08)	0.55
Exposure to COVID-19-specific information through different channels**												
Web-based media	1.04 (0.66-1.66)	0.86	1.32 (0.85-2.05)	0.21	0.71 (0.44-1.13)	0.15	0.84 (0.55-1.29)	0.44	1.41 (0.83-2.38)	0.20	1.23 (0.84-1.81)	0.28
Local channels	1.46 (0.98-2.18)	0.06	2.73 (1.78-4.20)	<0.001	1.28 (0.86-1.89)	0.22	2.05 (1.35-3.12)	0.001	1.63 (0.99-2.71)	0.06	1.37 (0.93-2.01)	0.11
Healthcare workers	1.47 (1.01-2.15)	0.04	1.83 (1.22-2.74)	0.003	1.99 (1.37-2.90)	<0.001	1.59 (1.08-2.34)	0.02	2.31 (1.46-3.67)	<0.001	1.75 (1.20-2.53)	0.003
Family members and friends	1.73 (1.19-2.53)	0.004	2.92 (1.93-4.43)	<0.001	2.10 (1.44-3.06)	<0.001	2.64 (1.77-3.93)	<0.001	2.94 (1.81-4.76)	<0.001	2.02 (1.39-2.93)	<0.001

AOR – adjusted odds ratios, CI – confidence interval, B-IPQ – brief illness perception questionnaire, B-IPQ – brief illness perception questionnaire, SD – standard deviation, PHQ-9 – patient’s health questionnaire-9, GAD-7 – generalised anxiety disorder-7

*Adjusted models controlled for significant background variables in **Table 3**.

†Adjusted models controlled for age, education level, monthly income, religion, toilet sharing, household size, COVID-19 diagnosis history and vaccination status.

‡Adjusted models controlled for age, education level, monthly income, piped water, toilet sharing, COVID-19 diagnosis history and vaccination status.

§Adjusted models controlled for age, marital status and piped water.

‖High/very high.

¶Agree/strongly agree.

**Sometimes/always.

Regarding compliance with hand washing/hygiene, perceiving COVID-19 as having a longer timeline (AOR = 1.10; 95% CI = 1.03-1.17 and AOR = 1.10; 95% CI = 1.02-1.17) was associated with higher odds of compliance in both slum and estate communities. Perceiving having more identity (AOR = 1.10; 95% CI = 1.03-1.19) and emotional effect (AOR = 1.10; 95% CI = 1.03-1.19) of COVID-19 were associated with higher odds only in the slum community, while having more concern about the disease (AOR = 1.22; 95% CI = 1.09-1.36) was associated with higher odds only in the estate community. Perceiving having high chances of close contact with people having COVID-19 (AOR = 1.62; 95% CI = 1.06-2.48 and AOR = 2.28; 95% CI = 1.43-3.62) was associated with higher odds of hand washing/hygiene in both communities while perceiving having high chances of contracting COVID-19 in the next 30 days (AOR = 2.03; 95% CI = 1.20-3.43) was associated with higher odds only in the estate community. Moreover, having depression symptoms (AOR = 1.03; 95% CI = 1.00-1.06 and AOR = 1.07; 95% CI = 1.03-1.11) was associated with higher odds of hand washing/hygiene in both communities while having anxiety symptoms (AOR = 1.04; 95% CI = 1.00-1.08) was significantly associated with higher odds only in the estate community. In addition, frequent exposure to COVID-19 information through health care workers (AOR = 1.99; 95% CI = 1.37-2.90 and AOR = 1.59; 95% CI = 1.08-2.34), and family members and friends (AOR = 2.10; 95% CI = 1.44-3.06 and AOR = 2.64; 95% CI = 1.77-3.93) were associated with higher odds of hand washing/hygiene in both communities while getting COVID-19 information from local channels (AOR = 2.05; 95% CI = 1.35-3.12) was associated with higher odds only in the estate community (**Table 4**).

Regarding compliance with social distancing, perceiving COVID-19 as having a more emotional effect (AOR = 1.13; 95% CI = 1.03-1.24 and AOR = 1.07; 95% CI = 1.00-1.15) was associated with higher odds of compliance in both communities, while having more concern about the disease (AOR = 1.20; 95% CI = 1.07-1.33) was associated with higher odds only in the estate community. Perceiving people infected with COVID-19 as having a high death rate (AOR = 2.03; 95% CI = 1.18-3.49) was associated with higher odds of social distancing only in the slum community, while perceiving COVID-19 would result in permanent bodily damage among infected people (AOR = 1.48; 95% CI = 1.02-2.15) was associated with higher odds only in the estate community. However, perceiving having a high chance of contracting COVID-19 in the next 30 days (AOR = 0.27; 95% CI = 0.10-0.69) was associated with lower odds of compliance with social distancing in the slum community. In addition, having depression (AOR = 1.05; 95% CI = 1.02-1.09 and AOR = 1.08; 95% CI = 1.05-1.12) and anxiety (AOR = 1.05; 95% CI = 1.00-1.09 and AOR = 1.08; 95% CI = 1.04-1.12) symptoms were associated with higher odds of social distancing in both slum and estate communities, similar to frequent exposure to COVID-19 information from health care workers (AOR = 2.31; 95% CI = 1.46-3.67 and AOR = 1.75; 95% CI = 1.20-2.53), and family members and friends (AOR = 2.94; 95% CI = 1.81-4.76 and AOR = 2.02; 95% CI = 1.39-2.93) (**Table 4**).

### Association of variables of interest with compliance with personal preventive measures against COVID-19 in the whole sample

Additional analysis was done to examine the factors associated with PPM practice in the overall sample without stratifying into community groups. Results indicate that the slum community had lower odds of hand washing/hygiene (AOR = 0.88; 95% CI = 0.66-0.98) and social distancing (AOR = 0.49; 95% CI = 0.36-0.65) compared to the estate community (Table S2 in the **Online Supplementary Document**).

## DISCUSSION

To our knowledge, this is one of the first comparative analyses of the underserved slum and better-off communities regarding compliance with COVID-19 PPMs. The study has important implications as it reflected on the differential practices and compliance with PPMs and associated factors in the slum and estate communities, considering that future infectious disease outbreaks may force the public to use PPMs again. Although COVID-19 is no longer considered a public health emergency, our study had implications for future outbreaks of other infectious diseases. The comparative approach used in this study allows for the assessment of inequities in PPMs practice in each group. Furthermore, the study was guided by relevant theories, used stratified sampling methods, and considered factors at different levels. All of these could contribute to literature and are strengths of this study.

The study showed that slum residents had lower levels of compliance with all COVID-19 PPMs compared to estate residents. Same situation could happen in future infectious disease outbreaks, which might increase the slum community's vulnerability to such infectious diseases. The observed difference in PPM compliance between slum and estate dwellers aligns with our hypothesis. Compared to estate residents, slum residents live in deprived settings, which are crowded places, making social distancing hardly possible [12-14]. Slum residents also have poor access to fresh, clean water, making hand washing/hygiene a challenge [15,36]. Moreover, slum residents have low income and this was worsened by the adverse economic impact of the pandemic, making buying face masks unaffordable and an extra economic burden [13,14,16]. All these may explain the observed pattern of slum residents having lower compliance with PPMs compared to their estate counterparts. Notably, results of hand hygiene indicate that although more estate dwellers achieved the desired level of personal hygiene, some slum dwellers had it too. This implies that health literacy flows through slums but it needs to be improved. In future infectious disease outbreaks, more attention should be given to slum communities.

The study also revealed that most slum residents knew that COVID-19 can be transmitted through faeces. The slum residents also perceived COVID-19 had a higher impact on their lives and with a longer timeline. However, slum residents were more likely to have depression symptoms compared to estate residents. In this study, since more slum residents shared toilets than estate residents (91.8% vs 77.2%), it is therefore likely that they pay more attention to the risk of COVID-19 transmission through faeces. The better knowledge of the disease may also be attributed to the awareness campaigns that were decentralised and extended to slum communities. This involved door-to-door campaigns and community visits, and using megaphones to educate people about the disease and its prevention practices [3]. Slum dwellers were inordinately affected by the COVID-19 restrictive measures since their livelihoods were the most affected [3,4], which could also explain the observed higher score of identity (i.e., how much the disease affects their life) among this vulnerable group. Moreover, this greater loss of income, livelihood and socioeconomic impact, makes economic-related mental distress inescapable [13], thus the observed higher mental distress. Slum residents also faced more difficulties in accessing information about COVID-19 statistics and had less exposure to COVID-19-specific information through web-based media compared to estate residents. Slum dwellers tend to have lower socioeconomic status including low education levels, which could negatively impact their health literacy [37,38], thus facing more difficulties accessing health information. Moreover, the lack of smartphones and limited access to the internet, in general, could explain why slum residents reported less exposure to COVID-19 information via web-based media. These inequalities in knowledge, mental health stress, and access to health information might exist in future infectious disease outbreaks, which might affect effective prevention and control.

Regarding factors associated with compliance with COVID-19 PPMs, various components of illness perception (B-IPQ) had significant associations (with higher odds) in both slum and estate communities. Perceiving COVID-19 to have more consequences, emotional effects and a longer timeline, and having a more identity of the disease were associated with higher odds of compliance with face mask use in both slum and estate communities. Regarding compliance with hand washing/hygiene and social distancing, only perceiving COVID-19 as having a longer timeline and more emotional effect were associated with higher odds of compliance in both communities, respectively. Our findings agree with previous studies that have also reported illness perception to positively affect adherence to COVID-19 PPMs [25,39]. These findings imply that illness perception, which entails the cognitive appraisal and personal understanding of disease [24], can significantly influence the public's psychological and behavioural responses (in this case compliance with PPMs) to the disease condition or pandemic. Notably, some unique patterns in the association of illness perception with PPMs compliance were observed in the two groups. Having more identity and emotional effects of COVID-19 were associated with higher odds of hand hygiene but only in the slum community. Slum residents in this study reported more identity and emotional effects of the pandemic, which might partly explain the finding. In addition, having more concern about the disease was associated with higher odds of all three PPMs (mask use, hand washing/hygiene and social distancing) but only in the estate community, a reason for which remains indeterminate and calls for further in-depth investigation. It is likely that illness perceptions would have similar effects on preventive behaviours in future infectious disease outbreaks. Modifying illness perceptions through health promotion might contribute to the effective prevention and control of future infectious disease outbreaks.

Study results indicate that perceived infection risk and severity of COVID-19 were associated with higher odds of hand washing/hygiene and social distancing in both communities. Perceived risk was also significantly associated with higher odds of face mask use but only in the estate community. Perceived threat, in general, has been reported to have a positive impact on compliance with COVID-19 PPMs in previous studies [40,41], which supports our findings. Mental distress, in terms of depression and anxiety symptoms, was associated with higher odds of compliance with all three COVID-19 PPMs in both communities. This is opposite to what we expected but supported by a previous study that also reported higher odds of COVID-19 PPM practice among those with mental distress/illness [42]. However, several other studies have reported opposite findings with less odds of PPMs practice and compliance among individuals with mental distress [26,27,43,44]. The possible reason for our finding may be that individuals who are psychologically bothered by the socio-economic impact of the pandemic would be more likely to take extra precautions to prevent further impact from COVID-19 infection, thus the observed higher compliance with PPMs.

### Implications of study findings

Our study findings have some practical implications for improving community practice and adherence to PPMs, and overall control of current and future infectious disease outbreaks. There is a need for more efforts in community health education and awareness of the importance of PPMs adherence to address the observed low compliance rates in both communities. Such educational strategies should focus on fostering positive knowledge and perceptions among the public since illness perception was a key determinant of PPMs compliance. In addition, COVID-19 or similar future pandemic control strategies should consider providing free or subsidised face masks, soap and sanitisers, especially to the disadvantaged urban poor. There is a need to address the water, sanitation, and hygiene needs of slum communities, for example, by ensuring access to safe and clean water, and improved toilet facilities, since lack of piped water and toilet sharing had a negative impact on PPMs compliance. This is essential for not only COVID-19 but also other preventable infectious diseases. Pandemic control programs also need to consider engaging religious leaders in PPM promotional campaigns, since religion was a significant determinant of PPMs compliance. Moreover, PPMs promotion strategies should consider using health care workers, family members and friends as key significant others and trusted sources of COVID-19 information, since frequent information exposure from these sources was associated with higher odds of PPMs compliance.

### Limitations of the study

The study had some limitations. There was a risk of information and recall bias as the study outcomes were self-reported and prone to social desirability, and this could affect the true estimation of PPMs practice in the sample. In addition, some measurement tools were constructed for this study, since there were no validated tools, although their reliability was acceptable. The slum and estate residents in this study were recruited in just two districts of Uganda, which affects the generalisability of the study results. Moreover, due to the cross-sectional design employed, inference and causation establishment are not possible beyond mere associations. Despite the limitations, the study provides useful insights into the differential practice of COVID-19 PPMs and associated factors in the underserved slum and better-off estate communities of Uganda.

## CONCLUSIONS

Although poor compliance was noted in both communities, the study found that slum communities had lower compliance with COVID-19 PPMs (mainly social distancing and hand washing/hygiene) compared to estate communities. To improve PPMs compliance in the current and future infectious disease outbreaks, the study results indicate a need for more programming and policy efforts in community education and sensitisation to address the poor PPMs compliance rates, and this should focus on promoting knowledge and improving perceptions of the disease. Addressing the water and sanitation needs of underserved slum communities is also vital in the control of not only COVID-19 but also other common infectious diseases. Moreover, providing free or subsidised face masks and soap to the most vulnerable, as well as engagement of religious leaders in pandemic control programs are yet other useful strategies.

## Additional material


Online Supplementary Document
