# Supplementary material for: Differential practice and associated factors of COVID-19 personal preventive measures among the slum and estate communities of Uganda: A community-based cross-sectional survey
Source: J Glob Health. 2023 Sep 15;13:06039. doi: 10.7189/jogh.13.06039 (PMC10502765; doi:10.7189/jogh.13.06039)
Supplement: Online Supplementary Document [file jogh-13-06039-s001.pdf]

Online Supplementary Material

Table S1. Association of variables of interest with compliance with personal preventive measures against COVID-19 among people from slum and estate communities

Variable	**Compliance with Mask use**				**Compliance with Hand washing/hygiene**				**Compliance with Social distance**			
	People from slum communities (n=511)		People from estate communities (n=514)		People from slum communities (n=511)		People from estate communities (n=514)		People from slum communities (n=511)		People from estate communities (n=514)	
	OR (95%CI)	P value	OR (95%CI)	P value	OR (95%CI)	P value	OR (95%CI)	P value	OR (95%CI)	P value	OR (95%CI)	P value
**Perceptions related to COVID-19**												
Brief illness representation (B-IPQ) related to COVID-19												
Consequences	1.06(1.00-1.12)	**0.04**	1.09(1.03-1.16)	**0.003**	1.03(0.98-1.10)	0.25	1.03(0.98-1.10)	0.25	1.07(0.99-1.15)	0.09	1.03(0.97-1.10)	0.30
Timeline	1.13(1.06-1.20)	**<0.001**	1.15(1.08-1.22)	**<0.001**	1.10(1.03-1.17)	**0.003**	1.10(1.04-1.18)	**0.002**	1.06(0.99-1.15)	0.11	1.08(1.01-1.15)	**0.02**
Personal control	1.01(0.96-1.07)	0.64	0.99(0.94-1.05)	0.83	1.00(0.94-1.05)	0.89	0.97(0.92-1.03)	0.29	0.95(0.89-1.01)	0.11	0.95(0.89-1.00)	0.06
Treatment control	1.04(0.97-1.11)	0.23	1.03(0.96-1.11)	0.37	1.02(0.95-1.09)	0.66	1.03(0.96-1.11)	0.41	0.97(0.89-1.05)	0.43	1.04(0.97-1.12)	0.29
Identity	1.11(1.04-1.19)	**0.003**	1.13(1.06-1.21)	**<0.001**	1.10(1.02-1.18)	**0.01**	1.03(0.96-1.09)	0.45	1.05(0.97-1.15)	0.22	1.06(0.99-1.13)	0.11
Coherence	1.04(0.98-1.12)	0.19	1.10(1.03-1.18)	**0.01**	0.96(0.90-1.03)	0.25	1.03(0.96-1.11)	0.37	0.99(0.91-1.07)	0.74	1.01(0.94-1.09)	0.70
Concern	1.03(0.94-1.12)	0.57	1.32(1.19-1.47)	**<0.001**	1.04(0.95-1.14)	0.43	1.21(1.10-1.33)	**<0.001**	0.99(0.88-1.10)	0.83	1.19(1.07-1.32)	**0.001**
Emotions	1.13(1.06-1.21)	**<0.001**	1.09(1.02-1.16)	**0.01**	1.09(1.02-1.17)	**0.01**	1.05(0.98-1.11)	0.15	1.13(1.03-1.24)	**0.01**	1.08(1.00-1.15)	**0.04**
Perceived susceptibility to COVID-19, n (%) high/very high												
In general, how high is	0.67(0.41-	0.12	1.48(0.90-	0.12	0.99(0.60-	0.97	1.85(1.13-	**0.01**	0.26(0.10-	**0.004**	1.35(0.83-	0.23

your chance of contracting COVID-19 in the next 30 days?	1.11)		2.42)		1.64)		3.02)		0.65)		2.22)	
How high is your chance of having close contact with people having COVID-19	1.27(0.85-1.92)	0.25	1.84(1.19-2.84)	**0.01**	1.54(1.02-2.32)	**0.04**	2.13(1.38-3.27)	**0.001**	0.90(0.53-1.53)	0.70	1.33(0.86-2.05)	0.20
Perceived severity of COVID-19, n (%) agree/strongly agree												
COVID-19 would result in permanent bodily damage among infected people	0.82(0.58-1.16)	0.27	1.35(0.95-1.91)	0.09	1.00(0.70-1.44)	0.98	0.99(0.70-1.40)	0.95	1.63(1.04-2.57)	**0.03**	1.44(1.00-2.07)	**0.04**
People infected with COVID-19 have a high death rate	0.97(0.67-1.41)	0.89	0.83(0.57-1.21)	0.33	1.22(0.83-1.80)	0.30	0.78(0.54-1.14)	0.20	2.15(1.26-3.65)	**0.01**	1.25(0.84-1.85)	0.26
**Knowledge related to COVID-19**												
Number of correct responses to knowledge related to COVID-19	1.04(0.90-1.18)	0.61	1.16(1.01-1.32)	**0.03**	1.02(0.89-1.17)	0.75	1.09(0.96-1.24)	0.19	0.95(0.81-1.13)	0.57	1.13(0.98-1.30)	0.09
**Mental health status**												
Depression symptoms (score of the PHQ-9 scale)	1.04(1.01-1.07)	**0.01**	1.07(1.04-1.11)	**<0.001**	1.03(1.00-1.06)	**0.04**	1.06(1.03-1.10)	**<0.001**	1.05(1.02-1.09)	**<0.001**	1.08(1.05-1.12)	**<0.001**
Generalized anxiety symptoms (score of the GAD-7 scale)	1.03(1.00-1.07)	**0.04**	1.05(1.01-1.09	**0.01**	1.01(0.98-1.05)	0.46	1.03(0.99-1.07)	0.09	1.04(1.00-1.08)	**0.04**	1.08(1.04-1.12)	**<0.001**
**Difficult to access COVID-19-related information**												
Difficult to Access	0.86(0.77-	**0.01**	0.96(0.86-	0.36	0.88(0.78-	**0.04**	0.90(0.80-	0.07	0.94(0.81-	0.41	0.96(0.85-	0.46

COVID-19 Information Scale	0.97)		1.07)		0.99)		1.01)		1.09)		1.07)	
**Exposure to COVID-19-specific information through different channels, n (%) sometimes/always**												
Web-based media	1.10(0.73-1.68)	0.65	1.85(1.28-2.67)	**<0.001**	0.70(0.45-1.08)	0.11	1.27(0.89-1.84)	0.19	1.33(0.80-2.21)	0.27	1.20(0.82-1.75)	0.34
Local channels	1.60(1.10-2.32)	**0.01**	2.87(1.98-4.16)	**<0.001**	1.28(0.87-1.88)	0.20	2.11(1.46-3.05)	**<0.001**	1.62(0.99-2.67)	0.06	1.35(0.93-1.96)	0.12
Healthcare workers	1.41(1.00-2.01)	**0.04**	1.76(1.24-2.51)	**<0.001**	1.89(1.32-2.71)	**<0.001**	1.50(1.05-2.12)	**0.02**	2.14(1.37-3.35)	**<0.001**	1.68(1.17-2.42)	**0.01**
Family members and friends	1.61(1.13-2.29)	**0.01**	2.91(2.03-4.16)	**<0.001**	1.93(1.35-2.78)	**<0.001**	2.75(1.92-3.94)	**<0.001**	2.74(1.72-4.38)	**<0.001**	2.02(1.40-2.91)	**<0.001**

OR: Unadjusted odds ratios, CI: Confidence interval, B-IPQ: Brief illness perception questionnaire

Table S2. Association of variables of interest with compliance with personal preventive measures against COVID-19 in the combined sample (N=1025)

Variable	Compliance with Mask use		Compliance with Hand washing/ hygiene		Compliance with Social distance	
	AOR (95%CI)*	P value	AOR (95%CI)**	P value	AOR (95%CI)***	P value
**Community status**						
Estate community	Referent category		Referent category		Referent category	
Slum community	0.86(0.64-1.15)	0.30	0.88(0.66-0.98)	**0.04**	0.49(0.36-0.65)	**<0.001**
**Perceptions related to COVID-19**						
Brief illness representation (B-IPQ) related to COVID-19						
Consequences	1.08(1.03-1.13)	**0.001**	1.04(1.00-1.09)	0.04	1.04(0.99-1.09)	0.11
Timeline	1.14(1.08-1.19)	**<0.001**	1.10(1.05-1.15)	**<0.001**	1.05(1.00-1.10)	**0.045**
Personal control	1.00(0.96-1.04)	0.99	0.98(0.94-1.02)	0.42	0.96(0.92-1.00)	0.05
Treatment control	1.04(0.98-1.09)	0.18	1.02(0.97-1.07)	0.49	1.01(0.95-1.07)	0.75
Identity	1.12(1.06-1.17)	**<0.001**	1.06(1.01-1.12)	**0.02**	1.04(0.98-1.09)	0.17
Coherence	1.05(1.00-1.11)	**0.045**	0.98(0.93-1.03)	0.44	1.02(0.96-1.07)	0.57
Concern	1.13(1.06-1.21)	**<0.001**	1.11(1.04-1.19)	**0.003**	1.09(1.01-1.18)	**0.02**
Emotions	1.11(1.06-1.17)	**<0.001**	1.07(1.02-1.12)	**0.01**	1.09(1.03-1.15)	**0.002**
Perceived susceptibility to COVID-19, high/very high						
In general, how high is your chance of contracting COVID-19 in the next 30 days?	1.03(0.72-1.48)	0.86	1.47(1.02-2.10)	0.04	0.83(0.55-1.24)	0.36
How high is your chance of having close contact with people having COVID-19	1.50(1.10-2.06)	**0.01**	1.89(1.39-2.58)	**<0.001**	1.15(0.83-1.60)	0.41
Perceived severity of COVID-19, agree/strongly agree						
COVID-19 would result in permanent bodily damage among infected people	1.04(0.80-1.35)	0.77	1.00(0.77-1.29)	0.98	1.53(1.15-2.03)	**0.003**
People infected with COVID-19 have a	1.04(0.78-1.38)	0.79	1.11(0.83-1.46)	0.48	1.57(1.15-2.15)	**0.01**

high death rate						
**Knowledge related to COVID-19**						
Number of correct responses to knowledge related to COVID-19	1.06(0.96-1.17)	0.24	1.02(0.92-1.13)	0.69	1.03(0.92-1.14)	0.63
**Mental health status**						
Depression symptoms (score of the PHQ-9 scale)	1.06(1.03-1.08)	**<0.001**	1.05(1.03-1.07)	**<0.001**	1.06(1.04-1.09)	**<0.001**
Generalized anxiety symptoms (score of the GAD-7 scale)	1.05(1.02-1.08)	**0.001**	1.03(1.00-1.06)	**0.02**	1.06(1.03-1.09)	**<0.001**
**Difficult to access COVID-19-related information**						
Difficult to Access COVID-19 Information Scale	0.95(0.88-1.03)	0.24	0.91(0.84-0.99)	**0.04**	0.95(0.86-1.04)	0.24
**Exposure to COVID-19-specific information through different channels, sometimes/always**						
Web-based media	1.21(0.89-1.64)	0.22	0.80(0.59-1.09)	0.16	1.44(1.07-1.96)	**0.02**
Local channels	1.92(1.45-2.53)	**<0.001**	1.62(1.23-2.15)	**0.001**	1.44(1.07-1.95)	**0.02**
Healthcare workers	1.55(1.19-2.01)	**0.001**	1.75(1.35-2.28)	**<0.001**	1.94(1.46-2.57)	**<0.001**
Family members and friends	2.04(1.56-2.67)	**<0.001**	2.30(1.76-3.02)	**<0.001**	2.27(1.70-3.02)	**<0.001**

Note: Adjusted models controlled for significant background variables in Table 3.
AOR: Adjusted odds ratios, CI: Confidence interval, B-IPQ: Brief illness perception questionnaire
*: Adjusted models controlled for age, education level, monthly income, religion, toilet sharing, household size, COVID-19 diagnosis history and vaccination status.
**: Adjusted models controlled for age, education level, monthly income, piped water, toilet sharing, COVID-19 diagnosis history and vaccination status.
***: Adjusted models controlled for age, marital status and piped water.
